# A Chicken Bone as an Unusual Cause of Bezoar-Induced Small Bowel Obstruction: A Case Report

**DOI:** 10.7759/cureus.23025

**Published:** 2022-03-10

**Authors:** Stephanie E Cornish

**Affiliations:** 1 Surgery, University of Queensland, Brisbane, AUS; 2 General Surgery, Toowoomba Base Hospital, Toowoomba, AUS

**Keywords:** foreign body, intraluminal abnormality, intraabdominal adhesions, chicken bone, small bowel obstruction, bezoar

## Abstract

Bezoars are a rare and often overlooked cause of small bowel obstruction, arising from an accumulation of indigestible foreign material, often being plant-based in origin. The majority of these cases arise in a predictable patient subgroup. The author presents an unusual case of a small bowel obstruction arising from a chicken bone encased in feculent material in a patient with no classical risk factors for bezoar formation.

## Introduction

Small bowel obstruction is a common surgical emergency caused by many pathological processes including adhesions (60-80% of cases in developed countries), volvulus, intussusception, hernia, and tumors [1-2]. However, it can be very difficult to clinically delineate the underlying cause of obstruction, as most present with the same compilation of symptoms and signs, including vomiting, nausea, abdominal pain, obstipation, fever, and an elevated leukocyte count [1].

A bezoar is a solidified substance formed by mixing indigestible exogenous materials with other substances in the gastrointestinal tract and is classified by composition into five subgroups; phytobezoar (plant material), trichobezoar (hair), pharmacobezoar (medication), lactobezoar (milk protein and mucus), and foreign body bezoars [3-4]. While uncommon as a whole, bezoars are associated with complications including ulceration, perforation, intussusception, and obstruction, and can cause mortality in up to 30% of cases [5]. Phytobezoars are by far the most frequently encountered [4]. The leading risk factor for bezoar formation is previous gastrointestinal surgery, due to either prevention of complete digestion caused by decreased gastric motility, or from an expanded stomach outlet leading to undigested fiber entering the small bowel more easily in other situations including postgastric resection, gastrojejunostomy, or pyloroplasty [1,4]. Other common causes include high vegetable-based diets, for example, persimmons and oranges in Spain or mushrooms in China are frequently found in bezoars in these countries, poor dentition leading to poor mastication of food, and metabolic diseases such as diabetes and hypothyroidism, likely resulting from gastoparesis [2,4,6]. Less commonly, abnormalities of the gastrointestinal tract itself cause narrowing and a resultant point of entrapment of the bezoar, including stenosing or stricturing secondary to Crohn’s disease or intestinal tuberculosis [1].

Research suggests that phytobezoar is the cause in 4.8-6% of cases of bowel obstruction, with 69-78% of these cases having had previous gastric surgery [4]. The bezoar usually becomes trapped in a narrow section of the small intestine, most commonly 50-70cm from the ileocecal junction where the bowel lumen narrows, although the site of obstruction depends on both the size of the bezoar and that of the lumen [1]. Furthermore, there is a documented increase in the incidence of bowel obstruction from bezoars in patients with mental disabilities, with surgical intervention for this obstruction accounting for 10% of all gastrointestinal operation in this subset of patients [4]. This increased risk in patients with mental disabilities is thought to be secondary to the increased propensity for their bezoars to be foreign body based, particularly plastic, paper, and worms, all of which are very difficult for the body to digest.

## Case presentation

**Figure 1:**
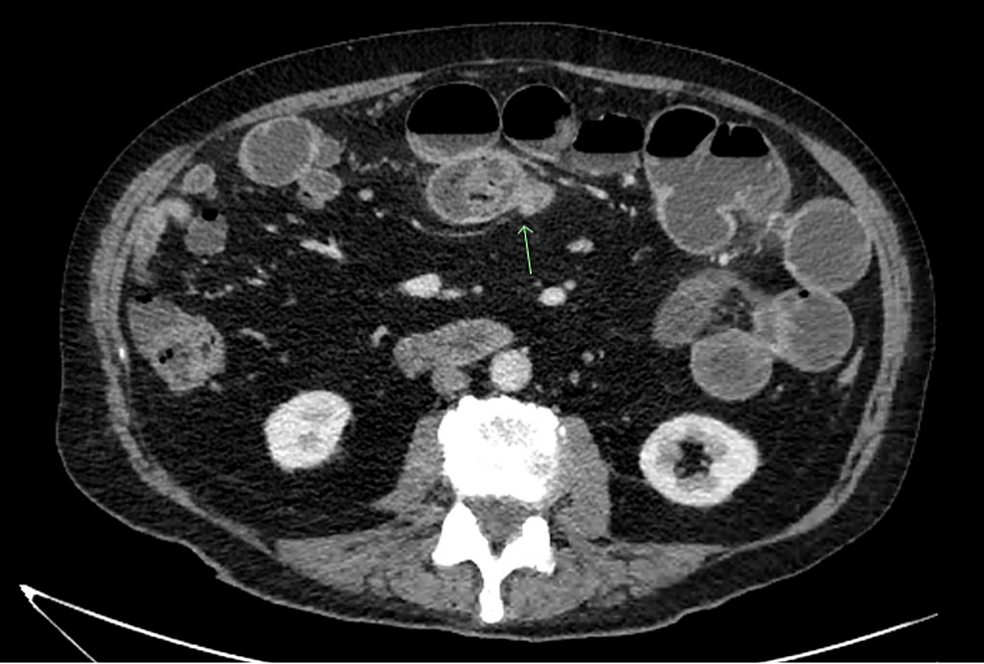
Contrast-enhanced CT scan demonstrating transition point of small bowel obstruction.

A 73-year-old male patient presented with abdominal pain that commenced eight days prior to presentation, intermittent vomiting, and reduced frequency of stools. He had undergone a radical prostatectomy for malignancy 15 years prior; however, he had no active medical conditions and particularly no risk factors for bezoar formation. He initially presented to a local general practitioner, who referred him for a computed tomography (CT) scan of the abdomen and pelvis, which demonstrated dilatation and water filling of proximal small bowel loops with a transition point in the center of the abdomen consistent with a small bowel obstruction (Figure 1). An incidental finding of multiple jejunal diverticuli in the right abdomen and diffuse colonic diverticuli was reported.

On presentation to the hospital, he was hemodynamically stable with no evidence of peritonitis. On hematological studies, white blood cell count was 16.8x10^9^/L (normal: 4.0-11.0x10^9^/L) with a hematocrit of 0.50 (normal: 0.39 - 0.52), while biochemically creatinine was elevated at 177umol/L (normal: 60-110umol/L) and C-reactive protein was also mildly elevated at 23mg/L (normal: <5mg/L).

**Figure 2:**
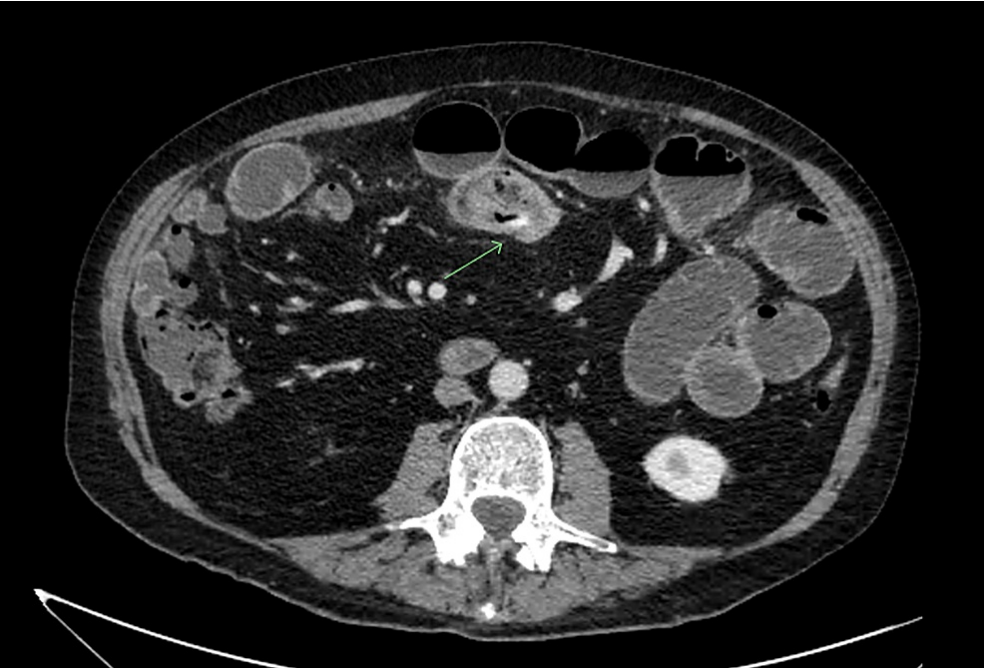
Contrast-enhanced CT scan again demonstrating a transition point but raising concerns of intraluminal abnormality such as intussusception or a foreign body.

The patient was admitted with a diagnosis of an early small bowel obstruction, presumed to be secondary to adhesions, and mild acute renal failure. Conservative management was undertaken as the patient was hemodynamically stable with nil evidence of peritonism, which would necessitate emergency surgical management at that time. Despite nasogastric tube placement and Gastrografin follow-through studies, the patient continued to have vomiting and high-volume, bilious nasogastric outputs that became feculent on day 3 of admission, and there was no discharge of flatus or feces. Original CT imaging was reviewed by the admitting surgeon, with concerns that the transition point identified may instead be reflective of an intussusception or foreign body rather than secondary to an adhesion as originally reported radiologically (Figure 2). While remaining hemodynamically stable, the failure to respond to conservative management necessitated that the patient proceeds to emergency surgery on day 4 of admission.

Diagnostic laparoscopy was performed with an open left upper quadrant entry for a 10mm port and two 5mm ports to the left side placed under vision. Inspection showed adhesions of the lower abdomen in keeping with known previous surgery; however, these were unrelated to the area of obstruction. At exploration, there was a change in small bowel caliber and the presence of a palpable intraluminal foreign body. An upper-midline mini-laparotomy was performed to facilitate externalization of offending length of the small bowel, and a longitudinal enterotomy of the small bowel performed over the foreign body. This foreign body was found to be a cylindrical bezoar 50mm in length and consisted of a 20mm chicken bone surrounded by hard feculent and fibrous material. The enterotomy was closed transversely using 3-0 PDS and a hand-sewn technique. No further masses were detected in the small bowel, and the multiple jejunal diverticuli proximal to the site of obstruction were soft.

Post-operatively, the patient progressed uneventfully and tolerated successive dietary upgrades. Flatus was passed on post-operative day 2 (POD 2), and a large bowel motion on POD 3, following which the patient was successfully discharged home the same day.

The patient had no recollection of eating chicken in the weeks preceding his admission. As such, it is speculated that the bone had been deposited at some point in the medium-to-distant past with progressive bezoar formation by insipient feculent matter, and subsequently mobilized intra-luminally causing obstruction of the small bowel at a narrower distal segment.

## Discussion

In the case put forward, the patient had none of the aforementioned risk factors for bezoar formation, with no history of gastrointestinal operation, metabolic disorders, or mental illness. Although he had undergone a radical prostatectomy, this would have involved minimal if any manipulation of the small bowel, which could potentially precipitate bezoar formation. Furthermore, the transition point of his obstruction was not at the level of a diverticulum, and as such the obstruction was not caused by stenosis as a result of diverticular disease. As for the bezoar itself, it was highly unusual in that the central nidus was a chicken bone and thereby animal-based, as opposed to the far more common phytobezoar that is plant-based. No other cases available in the literature have described an animal-based nidus.

Accurate pre-operative diagnosis of bezoar-induced small bowel obstruction is notoriously difficult [5]. X-ray, barium enema, and endoscopy can be used to clarify a diagnosis of bezoar-induced small bowel obstruction, but all offer poor diagnostic accuracy [1]. CT scanning is being used with increasing frequency in the diagnosis of this condition, with bezoars presenting as round or oval masses, often with well-coated envelopes, asymmetrical densities, and mottled gas densities inside the cavity [1]. The density varies depending on the contents of the bezoar, as does the presence of an envelope [3]. The benefit of CT in small bowel obstruction is the ability to demonstrate the site, degree, and likely cause of obstruction, as well as co-existing intestinal ischemia or potential intestinal diseases [3]. The sensitivity for detecting small bowel obstruction ranges from 73% to 95%, although sensitivity for bezoar-induced small bowel obstruction is speculated to be 83% in some studies and as low as 65% in others [2]. A retrospective study published by Wang et al. showed CT findings of a bezoar at the point of obstruction in all 35 patients [3], although as all patients proceeded to surgical intervention, this raises the possibility of confirmation bias on retrospective review of radiology.

In the case presented, none of the aforementioned “classical” features of bezoar were present radiographically. This led to a presumed diagnosis of a small bowel obstruction secondary to adhesions and attempted conservative management; however, evidence has shown that bezoar-induced small bowel obstruction usually requires early definitive operative therapy, as delays, particularly in the diagnostic phase, lead to greater morbidity [2].

However, other studies have shown that bezoar-based small bowel obstruction can be successfully managed with minimally invasive measures such as endoscopic retrieval, oral sodium bicarbonate solution, enzymatic digestion, or repeated gastric lavages, depending on anatomical location [1,6]. However, more recent studies advocate for prompt surgical management whether that be in the form of an exploratory (diagnostic) laparoscopy or laparotomy [6]. In the case of this patient, endoscopic treatment was not an option based on anatomical location, and attempts at lavage would not have been guaranteed given that the core of the bezoar was bone and hence could not be dissolved. Additionally, mobilizing the bone from its surrounding feculent material carries its own risks, with potential transition to distal small bowel and potential perforation, thus making operative management in this case the most appropriate option. Both laparoscopic and open approaches have been shown to be effective in the management of bezoars causing obstruction. Intra-operatively, with ever-increasing access to and skills with laparoscopic surgery, bezoars can now be managed with either extra-mural fragmentation and subsequent milking of the particulate matter to the caecum or with evisceration through a mini-laparotomy and enterotomy for particularly large or hard bezoars [7].

To the author’s knowledge, following a review of the available literature, this is the first case of bezoar-induced small bowel obstruction in a patient with no risk factors for classical bezoar formation and previously undiagnosed small bowel diverticular disease. It is furthermore believed to be the first case report of a bezoar to have an animal-based nidus for formation.

## Conclusions

While small bowel obstruction secondary to bezoar is well known to be a rare cause, the potential diagnostic difficulty posed by a non-specific constellation of clinical signs and highly variable radiographic sensitivity is further increased by cases of patients without known risk factors for bezoar formation. With known increased morbidity and mortality in delayed management of bezoar-induced obstructions, an appropriate index of suspicious must be held for this rare potential etiology, particularly in patients who are not progressing with conservative management.
